# Postoperative exercise rehabilitation and patient experience among breast cancer survivors under the “Healthy China” initiative: an integrative review

**DOI:** 10.3389/fpsyg.2025.1681492

**Published:** 2026-01-12

**Authors:** Binyu Zhao, Xuefei Li, Cong Fu, Ying Bian, Yafei Gong, Lina Zhu, Xiaochun Wang

**Affiliations:** 1 School of Nursing, Hebei University, Baoding, Hebei, China; 2 Department of Breast Surgery, Affiliated Hospital of Hebei University, Baoding, Hebei, China; 3 Department of Anesthesiology, Affiliated Hospital of Hebei University, Baoding, Hebei, China; 4 Department of Nursing, Affiliated Hospital of Hebei University, Baoding, Hebei, China

**Keywords:** breast cancer, exercise prescription, Healthy China, integrative review, postoperative rehabilitation

## Abstract

This integrative review synthesizes current evidence on postoperative exercise rehabilitation and patient experiences among breast cancer survivors, aiming to inform the development of scientific, standardized, and contextually appropriate rehabilitation protocols under the “Healthy China” initiative. A comprehensive literature search was conducted across seven Chinese and English databases, identifying 23 eligible studies published between 2015 and 2024, including randomized controlled trials and qualitative research. Methodological quality was assessed using the Mixed Methods Appraisal Tool (MMAT). The findings revealed substantial heterogeneity in exercise prescription parameters and a lack of unified guidelines. Exercise interventions were generally effective in improving physical function, reducing lymphedema risk, and enhancing psychological well-being, yet patient experiences varied considerably. Adherence was frequently hindered by treatment-related side effects, psychological resistance, and lack of individualized support. Combined interventions integrating multiple exercise modalities showed greater effectiveness than single-mode approaches. This review highlights the need for localized, evidence-based rehabilitation frameworks that consider patient preferences and provide psychological support throughout the recovery process. Nurses and case managers are encouraged to lead interdisciplinary teams in implementing structured exercise programs that include remote monitoring, individualized evaluation, and behavioral counseling to improve patient outcomes and long-term quality of life.

## 1 Introduction

Breast cancer is one of the most common malignancies among women (Sung et al., 2021). Although advances in comprehensive treatment have significantly extended survival, a range of postoperative complications continue to pose serious challenges to patients’ quality of life (Ghanei Gheshlagh et al., 2022). Among these, upper limb dysfunction and lymphedema are particularly prevalent, leading not only to reduced capacity for daily activities but also to considerable psychological distress, including anxiety and depression, which hinder patients’ return to normal life and social participation (Wu P. P. et al., 2023). Evidence shows that scientifically designed exercise interventions can effectively improve limb function and reduce the incidence of lymphedema (Feng et al., 2024; Liu et al., 2023). Domestic and international research has explored various forms of rehabilitation: resistance training to enhance muscle function by overcoming external resistance, such as using equipment and resistance bands (Li et al., 2018; Wu M. L. et al., 2023), aerobic exercise to improve cardiopulmonary endurance through sustained rhythmic activity, such as brisk walking or cycling (Dieli-Conwright et al., 2018; Huang et al., 2023), Flexibility training through stretching body parts to increase joint mobility (Liu et al., 2021), in addition, there are traditional Chinese sports that emphasize the unity of body and mind and slow movements, such as Baduanjin and Taijiquan (Liu et al., 2019; Wang et al., 2019). Under the “Healthy China 2030″ initiative, which emphasizes the integration of medicine and physical activity, studies have demonstrated that a 12-week exercise prescription can significantly enhance muscle strength and cardiopulmonary endurance in breast cancer patients (Liu et al., 2021). Furthermore, home exercise prescriptions based on “Internet+” technology—personalized workout plans created by healthcare professionals for patients through digital platforms like smart wearables and mobile apps, supported by remote monitoring and guidance—have been proven to significantly improve patients’ exercise adherence (Jiang et al., 2023).

However, significant challenges remain in the clinical application of exercise prescriptions. On one hand, there is no consensus on standardized implementation guidelines either domestically or internationally. Core parameters such as frequency, intensity, type, and duration vary widely across studies, leaving healthcare professionals without clear guidance when designing and implementing rehabilitation programs (Dieli-Conwright et al., 2018; Drozd et al., 2024). This lack of standardization compromises both the scientific basis and effectiveness of exercise interventions. On the other hand, common clinical issues such as insufficient individualization of exercise plans and poor patient adherence persist. Approximately 30% of patients report fear of exercise (Chang et al., 2024), and treatment-related side effects such as fatigue and pain further reduce patients’ motivation and continuity in participating in exercise rehabilitation, significantly limiting its benefits (Chen et al., 2022).

Due to significant heterogeneity in existing literature regarding exercise intervention protocols, outcome measures, and study designs, the data cannot be subjected to clinically meaningful meta-analysis. Therefore, this study adopted the integrative review method proposed by Whittemore and Knafl (2005), and guided by the PICOS framework, systematically explored the components of exercise prescriptions for postoperative breast cancer patients and their patient experience.

This review is guided by the central question: How to create localized exercise prescriptions and support strategies in China to better improve the exercise experience of Chinese breast cancer postoperative survivors? By synthesizing evidence from multiple sources, this review aims to provide a comprehensive understanding of the current state of exercise rehabilitation therapy for breast cancer patients after surgery. Ultimately, the goal is to support the development of a scientific, standardized, and localized rehabilitation framework tailored to China’s context, thereby improving recovery outcomes for breast cancer patients and advancing the goals of the “Healthy China” initiative.

It is worth noting that the “Healthy China 2030” initiative, with its core principle of integrating medicine and physical activity, aligns with the goals of the World Health Organization’s’ Global Action Plan for Physical Activity 2018–2030, which also advocates for incorporating physical activity into healthcare systems to prevent and manage non-communicable diseases. Although this study is rooted in the China context, it addresses a universal challenge in cancer rehabilitation. Therefore, the insights generated by this study can provide valuable references for developing culturally adapted rehabilitation models in other global healthcare settings.

## 2 Materials and methods

This integrative review was conducted following the methodology outlined by Whittemore and Knafl (2005) and adhered to the Preferred Reporting Items for Systematic Reviews and Meta-Analyses (PRISMA) guidelines to ensure a transparent, reproducible, and credible review process, thereby providing methodological assurance for the scientific conclusions.

We selected the integrative review method for its ability to synthesize diverse types of evidence—both quantitative and qualitative—within a single review. Unlike systematic reviews that typically focus on quantitative data and meta-analysis, or scoping reviews that aim to map the extent of research in a field, the integrative review allows for a comprehensive understanding of complex phenomena by integrating findings from varied study designs. Given that our research question encompasses both the effectiveness of exercise interventions (quantitative evidence) and the subjective experiences of patients (qualitative evidence), the integrative review provided a suitable framework to address both aspects holistically.

### 2.1 Scope of the study

The research question was formulated according to the PICOS framework to guide the review. The population (P) of interest comprised adult women following surgery for breast cancer. The intervention (I) was defined as any structured, postoperative exercise program or prescription. These interventions were evaluated in comparison (C) to routine care (e.g., standard physiotherapy or educational advice) or other non-exercise interventions. The outcomes (O) of primary interest were upper limb function, the incidence of lymphedema, and health-related quality of life. Finally, eligible study designs (S) included randomized controlled trials, quasi-experimental studies, and qualitative research, in order to synthesize both quantitative efficacy and qualitative experiential evidence.

### 2.2 Literature search strategy

A systematic literature search was conducted from inception until December 30, 2024, across seven electronic databases: PubMed, Web of Science, the Cochrane Library, CINAHL, China National Knowledge Infrastructure (CNKI), Wanfang Data, and VIP Chinese Journal Database (VIP). The search strategy was developed using a combination of Medical Subject Headings (MeSH) terms and free-text keywords related to three core concepts: “breast neoplasms,” “postoperative period,” and “exercise therapy.” The search strategy was tailored to the specific syntax of each database. The full search strategies are provided in Supplementary File 1.

### 2.3 Inclusion and exclusion criteria

Inclusion criteria:(1) Participants were adult women after breast cancer surgery; (2) The intervention involved a structured exercise program; (3) Study types included original research (e.g., randomized controlled trials, quasi-experimental studies, qualitative studies).

Exclusion criteria:(1) Studies with unclear descriptions of the exercise intervention protocol; (2) Publication types such as letters, conference abstracts, commentaries, or reviews; (3) Non-English or non-Chinese publications; (4) Duplicate publications or studies with highly overlapping content; (5) Studies for which the full text was unavailable.

Rationale for language restriction: The exclusion of publications in languages other than English or Chinese was a practical decision based on the research team’s language proficiency and the availability of translation resources. We acknowledge that this may introduce a potential for language bias by omitting relevant studies published in other languages. However, given that the major international (English) and the most relevant regional (Chinese) evidence bases were comprehensively searched, we believe the retrieved literature adequately represents the current state of knowledge on this topic, particularly within the context of the ‘Healthy China’ initiative.

### 2.4 Literature screening and quality appraisal

Literature management and screening were performed using Zotero 7. Following prior team training in systematic review methods, two researchers independently executed the study selection and quality appraisal processes. The literature screening (kappa = 0.86) and methodological assessment using the Mixed Methods Appraisal Tool (MMAT; kappa = 0.83) were conducted independently, with any discrepancies resolved through discussion or arbitration by a third researcher. For the MMAT, each study was rated on specific criteria, and a total quality score (percentage of criteria met) was calculated. The MMAT scores for all included studies are presented in Table 1.

**Table 1:** Basic characteristics of included studies.

No.	Author(s)	Title	Year	Country	Sample size	Study design	Quality score
1	Liu et al. (2021)	Design and implementation of exercise prescription to prevent upper limb lymphedema in breast cancer survivors	2021	China	106	Quasi-experimental (non-randomized)	100%
2	Son et al. (2024)	Effects of forearm resistance exercises on breast cancer-related lymphedema using segmental bioelectrical impedance analysis: a pilot RCT	2024	Korea	18	Randomized controlled trial	80%
3	Esteban-Simon et al. (2023)	Does a resistance training program affect between-arms volume difference and shoulder disabilities in female breast cancer survivors? secondary outcomes of the EFICAN trial	2023	Spain	60	Randomized controlled trial	100%
4	Jiang et al. (2023)	Application of “Internet+” home-based exercise prescription in postoperative rehabilitation of breast cancer patients	2023	China	117	Randomized controlled trial	100%
5	Lozano-Lozano et al. (2020)	Mobile health and supervised rehabilitation versus mobile health alone in breast cancer survivors: RCT	2020	Spain	80	Randomized controlled trial	100%
6	Jin et al. (2018)	Design and application of a virtual reality rehabilitation system for postoperative breast cancer patients	2018	China	76	Randomized controlled trial	100%
7	Xu and Wang (2021)	Application of “shoulder joint eight movements” in postoperative functional training for breast cancer patients	2021	China	102	Randomized controlled trial	100%
8	Paolucci et al. (2021)	The recovery of reaching movement in breast cancer survivors: two different rehabilitative protocols in comparison	2021	Italy	66	Randomized controlled trial	100%
9	Husebø et al. (2015)	Exercise: a path to wellness during adjuvant chemotherapy for breast cancer?	2015	Norway	27	Qualitative study	100%
10	Wechsler et al. (2023)	Finding the optimal exercise dose while living with cancer-related fatigue: a qualitative study	2023	USA	11	Qualitative study	100%
11	Tsai et al. (2018)	Physical activity and exercise self-regulation in cancer survivors: a qualitative study	2018	USA	35	Qualitative study	100%
12	Nielsen et al. (2020)	Preferences for mHealth physical activity interventions during chemotherapy for breast cancer: a qualitative evaluation	2020	USA	30	Qualitative study	100%
13	Leclerc et al. (2017)	Multidisciplinary rehabilitation program after breast cancer: benefits on physical function, anthropometry, and quality of life	2017	Belgium	209	Non-randomized controlled trial	100%
14	Drozd et al. (2024)	Post-cancer rehabilitation: multidisciplinary exercise program organization and feasibility	2024	France	655	Retrospective review	100%
15	Dieli-Conwright et al. (2018)	Effects of aerobic and resistance exercise on metabolic syndrome, sarcopenic obesity, and circulating biomarkers in overweight or obese breast cancer survivors: a randomized controlled trial	2018	USA	100	Randomized controlled trial	100%
16	Kilbreath et al. (2020)	Reduction of breast lymphedema secondary to breast cancer: a randomized controlled exercise trial	2020	Australia	88	Randomized controlled trial	100%
17	Đorđević et al. (2024)	The impact of online yoga on sleep and quality of life in women with breast cancer: a randomized trial	2024	Germany	173	Randomized controlled trial	100%
18	Rasmussen et al. (2022)	The analgesic effect of resistance training after breast cancer (ANTRAC): a randomized controlled trial	2022	Denmark	20	Randomized controlled trial	80%
19	Hiraoui et al. (2022)	Effects of a multimodal training program on muscle deoxygenation in women with breast cancer: a randomized controlled trial	2022	France	32	Randomized controlled trial	80%
20	Wang et al. (2019)	The health effects of Baduanjin exercise in breast cancer survivors in China: a randomized, controlled, single-blind trial	2019	China	86	Randomized controlled single-blind trial	100%
21	Scott et al. (2020)	Effects of exercise therapy dosing schedule on impaired cardiorespiratory fitness in patients with primary breast cancer: a randomized controlled trial	2020	USA	174	Randomized controlled trial	100%
22	Min et al. (2024)	Early implementation of exercise to facilitate recovery after breast cancer surgery: a randomized clinical trial	2024	Korea	56	Randomized controlled trial	100%
23	Sun et al. (2024)	Application of a progressive exercise prescription in breast cancer patients undergoing postoperative radiotherapy	2024	China	132	Non-randomized controlled trial	100%

### 2.5 Data extraction and analysis

This study adopts the strategy of thematic aggregation and interpretative integration, and regards the quantitative and qualitative evidence as complementary evidence body, which is equally weighted to construct the overall interpretation of the research problem. Extracted data included basic study characteristics (author, year, country/region), exercise prescription parameters (frequency, intensity, type, duration, total volume, and progression), study conclusions, and themes with supporting data in qualitative studies. In this study, qualitative data analysis was independently conducted by two researchers using NVivo 12.0 software for data management, coding, and theme extraction. A combination of inductive and deductive coding methods was employed to systematically analyze raw data, with continuous refinement of coding definitions until no new codes emerged and data saturation was achieved. Disputes during coding were resolved through bilateral discussions, with a third researcher intervening if consensus remained unattained. Cohen’s Kappa coefficient (Kappa = 0.82) demonstrated strong intercoder consistency, indicating high reliability in the analysis.

## 3 Results

### 3.1 Literature search results

**Figure 1:**
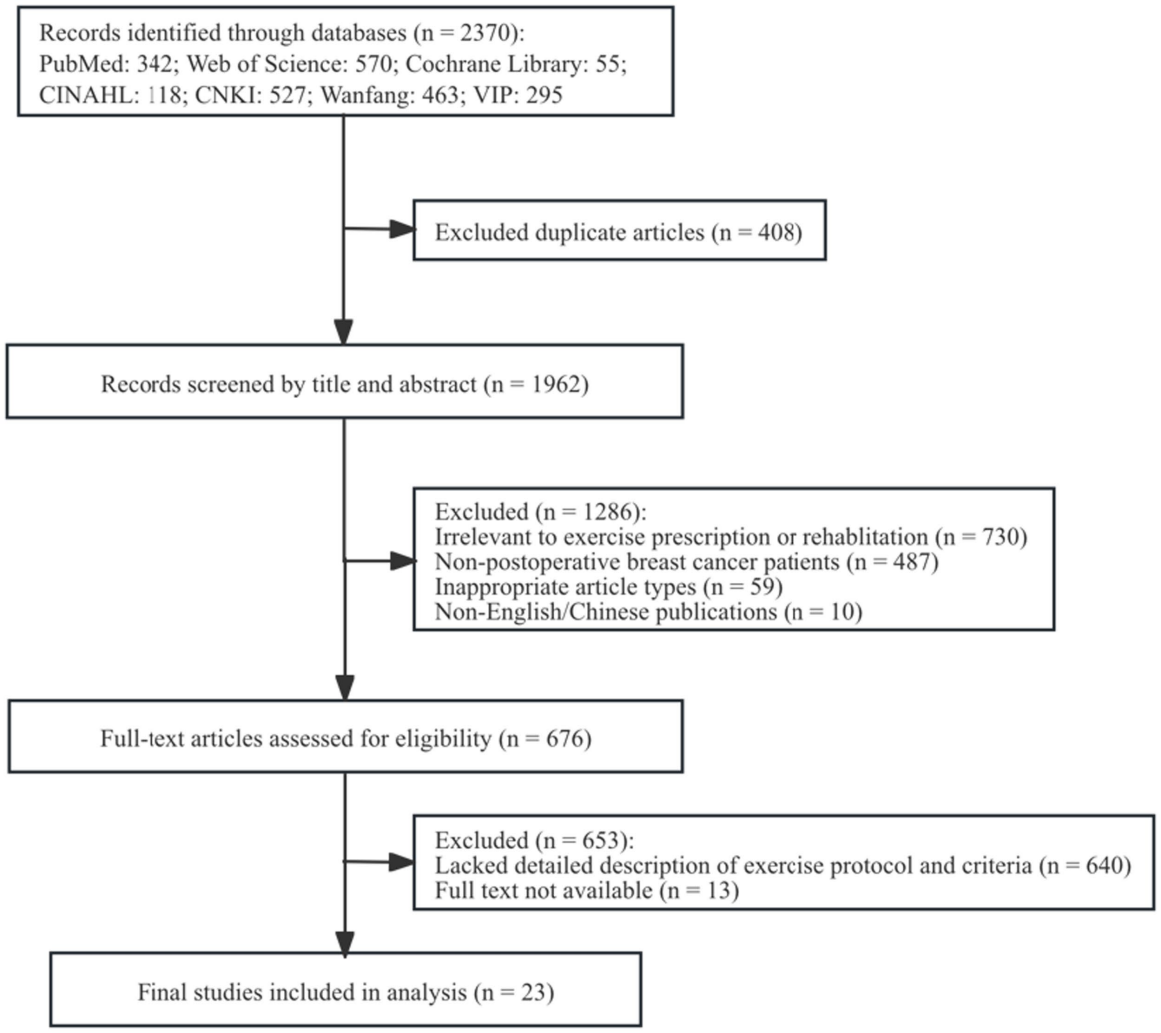
PRISMA flow diagram of literature screening.

A total of 2,370 records were initially retrieved from the databases, including 342 from PubMed, 570 from Web of Science, 55 from the Cochrane Library, 118 from CINAHL, 527 from CNKI, 463 from Wanfang Data, and 295 from VIP. After removing duplicates and screening titles, abstracts, and full texts, 23 studies were ultimately included. The methodological quality was assessed using the Mixed Methods Appraisal Tool (MMAT). The overall quality of the evidence was high, with 20 studies scoring 100% and 3 studies scoring 80% (Hiraoui et al., 2022; Rasmussen et al., 2022; Son et al., 2024), providing a reliable foundation for the subsequent synthesis of results. The literature selection process is presented in Figure 1.

### 3.2 Methodological quality of included studies

The methodological quality of the 23 included studies was assessed using the Mixed Methods Appraisal Tool (MMAT), with detailed scores for each study provided in Table 1 and Supplementary File 2. The overall quality of the evidence was high. Twenty studies (87%) fulfilled all MMAT criteria, achieving a score of 100%. The remaining three studies (Hiraoui et al., 2022; Rasmussen et al., 2022; Son et al., 2024) met 80% of the criteria. No studies were excluded based on quality assessment, all were deemed to provide valuable evidence for this integrative review. The high methodological quality across the included literature provides a reliable foundation for the subsequent synthesis of results.

### 3.3 Basic characteristics of included studies

Among the 23 included studies, 15 were randomized controlled trials (RCTs), 3 were non-randomized controlled trials, 4 were qualitative studies, and 1 was retrospective. Six studies were conducted in China, while the remaining 17 were from countries such as the United States, France, and Germany. The included studies were published between 2015 and 2024. Detailed characteristics of the included studies are presented in Table 1.

As shown in Table 1, the literature included in this study exhibits significant methodological heterogeneity across multiple dimensions, including research design, exercise intervention protocols, participant characteristics, and outcome measurement indicators. The most prominent source of heterogeneity stems from substantial variations in core parameters such as exercise prescription types, intensity levels, and frequency. The diversity of exercise prescription interventions highlights the urgent need for integrated analysis.

### 3.4 Core principles of postoperative exercise prescription for breast cancer patients

Table 2 systematically summarizes the core elements, implementation processes, and intervention outcomes of postoperative exercise prescriptions for breast cancer patients based on the PICOS framework. From the integrated results of 23 studies, it is clear that the core composition dimensions of postoperative exercise prescriptions for breast cancer patients are well-defined, covering six key aspects: exercise frequency, intensity, duration, type, total volume, and progression methods. These dimensions collectively form the core framework of postoperative exercise prescription intervention, providing a fundamental reference for the clinical development of targeted exercise prescriptions. However, considerable variability exists across studies in how these principles are defined and applied. Exercise frequency is expressed in diverse ways, such as a fixed number of sessions per week or a total number of minutes per week, with no unified standard. This inconsistency makes it difficult to compare the effects of frequency on rehabilitation outcomes across studies. Exercise intensity indicators are complex and differ between aerobic and resistance training. For example, methods such as heart rate reserve (HRR) and the Borg Rating of Perceived Exertion (RPE) scale are commonly used, but their variability increases the difficulty of determining appropriate intensity levels in clinical settings. Exercise duration is inconsistently reported, with some studies using minutes per session and others using hours per week, making it challenging to accurately assess the impact of exercise duration on outcomes. Total exercise volume is also quantified in various ways, including MET-min/week, kcal/week, and minutes/week. The lack of consensus on the most effective unit hampers the evaluation of the relationship between exercise volume and rehabilitation efficacy. In addition, descriptions of exercise progression are often vague or absent, limiting the ability to implement and adjust exercise prescriptions scientifically. Overall, these inconsistencies reflect a lack of standardization and normativity in the application of core principles in postoperative exercise prescription for breast cancer patients. This hinders the development of evidence-based, standardized rehabilitation protocols.

**Table 2:** Core components of postoperative exercise prescriptions for breast cancer patients.

Author	Population	Intervention	Comparison	Outcome	Study design
Liu et al. (2021)	Post-operative patients with breast cancer; Intervention group (*n* = 56) received guided exercise prescription.	*Frequency*: Aerobic: multiple sessions/week; Resistance: 2x/week; Flexibility: 2-3x/week *Intensity*: Aerobic: moderate-to-low, using HRR (40–60% of HRR); Resistance: 40–50% 1RM progressing to 50–70% 1RM *Duration*: At least 150 min/week; Each session includes 5–10 min warm-up, main workout, and 5–10 min cool-down *Type*: Aerobic: brisk walking, jogging, aerobics, cycling, dancing, swimming; Resistance: wall push-ups, chest presses, lateral raises, arm curls, squats, leg lifts, etc.; Flexibility: stretching of upper/lower limbs and torso muscles *Total Volume*: Aerobic: ≥500–1,000 MET-min/week; Resistance: intensity adjusted progressively via 1RM; Flexibility: 3–60 s/stretch, 2–4 reps Progression: Weeks 1–4: Increase duration by 5–10 min every 1–2 weeks, frequency by 1–2 sessions, and intensity by 5–10%; Resistance training progressed from 40–50% to 50–70% 1RM	Control group (*n* = 50) received routine health education.	The upper limb strength and cardiopulmonary endurance were improved, and the incidence of lymphedema was reduced.	Quasi-experimental study (non-randomized).
Son et al. (2024)	Patients with unilateral breast cancer-related lymphedema; Intervention group (*n* = 10) received resistance training.	*Frequency*: 5 sessions/week for 2 weeks (10 sessions total) *Intensity*: Intervention: 60% 1RM (Week 1); Control: not specified *Duration*: 20 min/session: 5 min warm-up, 10 min resistance training, 5 min cooldown *Type*: Intervention: forearm resistance using resistance bands and handgrip exercises; Control: neck, shoulder, arm, wrist stretches + deep breathing. *Total Volume*: 100 min/week *Progression*: Progressed to 80% 1RM in Week 2	Control group (*n* = 8) received stretching exercises.	Resistance training of forearm may be effective in the management of lymphedema	Randomized controlled trial.
Esteban-Simon et al. (2023)	Post-operative patients with breast cancer; Intervention group (*n* = 32) received resistance training.	*Frequency*: 2 sessions/week *Intensity*: Intensity progressively increased *Duration*: 50 min/session: 10 min warm-up, 30 min resistance training, 10 min cooldown *Type*: Preparation section: Aerobic activities, thoracic mobility training, core stability training, scapular and shoulder joint stability training. Resistance training section: including unilateral isometric seated bench press, unilateral isometric seated row training. Relaxation section: Stretching exercises *Total Volume*: 12 weeks *Progression*: Gradual increase in intensity and load over the intervention period	Control group (*n* = 28) received routine care.	Resistance training may improve shoulder function in breast cancer survivors.	Randomized controlled trial.
Jiang et al. (2023)	Post-operative patients with breast cancer; The intervention group (*n* = 58) received the “Internet +” home exercise prescription program through smart wearable devices and via the WeChat public platform.	*Frequency*: 3–5 sessions/week *Intensity*: Progressed from light (40% HRmax) to moderate (60% HRmax) intensity; monitored via Borg RPE (9–13 = light to moderate) *Duration*: Each exercise session lasts for 30 to 50 min, including 5 to 10 min of warm-up, at least 3 to 5 days of aerobic exercise per week, 30 to 50 min per session, 2 times of strength training per week with an interval of more than 48 h, 2 to 3 times of stretching exercises per week, and 5 to 10 min of cool-down activities. *Type*: Aerobic (e.g., brisk walking, jogging, swimming), resistance (e.g., front/side arm raises, squats), flexibility (e.g., neck/shoulder stretches) Total Volume:12 weeks Progression: Every 1–2 weeks: increase duration by 5–10 min and intensity by 5–10%, progressing from light to moderate	Control group (*n* = 59) received routine care.	The “Internet+” home-based exercise prescription is convenient, visual, and user-friendly, significantly improving adherence and reducing the incidence of upper limb lymphedema while enhancing shoulder mobility.	Randomized controlled trial.
Lozano-Lozano et al. (2020)	Post-operative patients with breast cancer; Intervention group (*n* = 40) received mHealth + supervised rehab.	*Frequency*: 3 sessions/week *Intensity*: Intervention: moderate to high; Control: moderate *Duration*: 60 min/session (supervised rehab) *Type*: Symptom management, therapeutic exercise (joint mobility, strength training), psychomotor group activities *Total Volume*: 8 weeks *Progression*: Intervention: adjusted based on patient response; Control: feedback and suggestions based on patient records	Control group (*n* = 40) received mHealth only.	It is superior to the simple mobility health program in improving quality of life, upper limb function and symptom management.	Randomized controlled trial.
Jin et al. (2018)	Patients with unilateral or bilateral modified radical mastectomy; Intervention group (*n* = 38) received VR-based rehab.	*Frequency*: 2 sessions/day *Intensity*: Intervention: VR system adjusted resistance and intensity based on recovery stage and ability; Control: traditional rehab, intensity not specified *Duration*: 15–30 min/session *Type*: Intervention: upper-limb rehab device, motion-sensing equipment, 3D video guidance; Control: nurse-led demonstrations and rehab exercise videos *Total Volume*: 3 months *Progression*: Intervention: auto-adjustment of resistance and difficulty based on patient recovery stage; Control: no detailed progression specified	Control group (*n* = 38) received traditional rehab exercises.	VR-assisted rehabilitation significantly improved shoulder, increased training adherence, and reduced limb edema.	Randomized controlled trial.
Xu and Wang (2021)	Post-operative patients with breast cancer; Intervention group (*n* = 50) used “Shoulder Joint Eight Movements.”	*Frequency*: 3 sessions/week (Mon, Wed, Fri), twice daily *Intensity*: Gradually increased *Duration*: 30 min/session *Type*: “Shoulder Joint Eight Movements” – a qigong-based program combining Tai Chi, Baduanjin, Wuqinxi, and the “328 movement model” The exercise mainly involves gentle, slow, soft, smooth and continuous movements. The movements are smooth and fluid, and it targets the muscles in the arms, shoulders, chest, waist and legs. *Total Volume*: 12 weeks *Progression*: Initiated on postoperative day 11; gradually increased range and intensity	Control group (*n* = 52) received conventional rehab.	The patients’ compliance was improved, the upper limb function was restored, and the pain, daily activity ability, range of motion and muscle strength were improved.	Randomized controlled trial.
Paolucci et al. (2021)	Post-operative patients with breast cancer; The intervention group (*n* = 33) received individualized aerobic exercise and resistance exercise	*Frequency*: 2 sessions/week *Intensity*: Single rehab group: progressive intensity; Group rehab: moderate intensity *Duration*: 60 min/session *Type*: Single rehab: scapulothoracic myofascial mobilization and pectoral stretching; Group rehab: general basic exercises *Total Volume*: 6 weeks *Progression*: Single rehab group had increasing intensity and complexity, especially scapula-targeted; Group rehab: fixed content, no clear progression	The control group (*n* = 33) received group aerobic exercise and resistance training.	The pain was relieved and the function of upper limb was improved. The individualized rehabilitation showed better performance in the quality of movement.	Randomized controlled trial.
Leclerc et al. (2017)	Post-operative patients with breast cancer; Intervention group (*n* = 103) received 3-month rehab.	*Frequency*: Physical: 3x/week (90 min); Psychoeducation: 1x/week (2 h) *Intensity*: 60–70% MAP (cardio), 30–35% 1RM (resistance) *Duration*: ~4.5 h/week physical training + 2 h/week psychoeducation *Type*: Cardio (cycling), resistance training (upper/lower body), core work, Swiss ball, and other varied exercises *Total Volume*: 3 months *Progression*: Gradual increase in intensity and difficulty of physical training	Control group (*n* = 106) received no intervention	Multidisciplinary rehabilitation programs have achieved significant improvements in most aspects of physical function, body composition, and quality of life.	Non-randomized controlled trial.
Drozd et al. (2024)	Post-operative patients with breast cancer; (*n* = 655)	*Frequency*: ≥6 sessions/week, 45 min each *Intensity*: Borg RPE scale target 11–15 (light to moderate) *Duration*: ~4.5 h/week *Type*: Aerobic endurance training (cycle ergometer, treadmill, elliptical, outdoor walking); resistance training (machines, resistance bands, dumbbells) *Total Volume*: 14 weeks *Progression*: Exercise intensity and content were progressively adjusted based on patient’s physical condition to support adherence	Control group (*n* = 2,373) received no intervention	The program is feasible, sustainable and delivers positive treatment outcomes for a large number of patients	Retrospective review.
Dieli-Conwright et al. (2018)	100 overweight or obese Post-operative patients with breast cancer; the intervention group (*n* = 50) received a combined intervention.	*Frequency*: 3 sessions/week *Intensity*: Moderate to vigorous (65–85% of HRmax) *Duration*: ~80 min (Day 1 & 3), ~50 min (Day 2); ~240 min/week *Type*: Aerobic (treadmill, rowing machine, stationary bike); Resistance (leg press, squats, chest press, etc.) *Total Volume*: 16 weeks *Progression*: Intensity and/or duration increased every 4 weeks by adjusting repetitions or workload	The control group (*n* = 50) received only standard care	Significantly improved metabolic syndrome, sarcopenic obesity, and related biomarkers in overweight or obese breast cancer survivors.	Randomized controlled trial.
Kilbreath et al. (2020)	Women with stable breast cancer-related lymphedema; Exercise group (*n* = 41) Engage in aerobic and resistance training.	*Frequency*: 3 sessions/week *Intensity*: Aerobic: 60–85% HRR; Resistance: 10–12 RM, the intensity is adjusted according to the patient’s self-perceived level of fatigue. *Duration*: ~3 h/week *Type*: Low-intensity warm-up stretching exercises, 30-min resistance-building strength training (using free weights and variable resistance equipment), and two 10-min aerobic sessions (using stationary bicycles, treadmills, rowing machines or elliptical machines) *Total Volume*: 12 weeks *Progression*: Exercise content changed every 4 weeks to enhance engagement; intensity tailored to participant capacity	Control group (*n* = 47) received routine care.	Aerobic and resistance training program was safe for patients with secondary breast cancer-related lymphedema and significantly reduced symptoms and physical indicators without exacerbating swelling.	Single-blind randomized controlled
Đorđević et al. (2024)	Post-operative patients with breast cancer; Intervention group (*n* = 116) Provide yoga training	*Frequency*: 2 sessions/week *Intensity*: Moderate (basic yoga poses) *Duration*: 45 min/session *Type*: Online yoga: breathing (10 min), basic poses (20–30 min), meditation/relaxation (10 min) *Total Volume*: 6 weeks *Progression*: No specific progression; sessions are designed to suit both beginners and experienced participants	Control group (*n* = 57) maintained regular lifestyle.	Online yoga is a safe, low-cost, and easily implementable intervention that effectively improves dyspnea, physical activity levels, and sleep quality.	Randomized controlled trial.
Rasmussen et al. (2022)	Post-operative patients with breast cancer; Intervention group (*n* = 10) Engage in resistance training.	*Frequency*: 2 sessions/week *Intensity*: Start at 60% 1RM; adjusted by performance; 3 stages per session: 2–4 sets × (10–12, 6–8, 2–4 reps) *Duration*: ~1 h/session *Type*: Progressive full-body resistance training: box squats, bench press, dumbbells, rows, lat pulldowns *Total Volume*: 12 weeks *Progression*: Training volume and intensity are adjusted every 4 weeks to increase load gradually	Control group (*n* = 10) Maintain a normal standard of living.	The pain threshold and maximum muscle strength were increased, and the mechanical pain sensitivity was decreased.	Randomized controlled trial.
Hiraoui et al. (2022)	Post-operative patients with breast cancer who have received adjuvant chemotherapy; Intervention group (*n* = 20) received multimodal training.	*Frequency*: 5x/week: home walking & isometric training; 2x/week: aerobic cycling; 2x/week: electrical muscle stimulation training *Intensity*: Aerobic: 55–80% THRmax; Isometric: 10 × 3 s → 20 × 5 s; electrical muscle stimulation: intensity based on tolerance *Duration*: Walking: 20 min; Cycling: from 10 min *Type*: Interval aerobic cycling, home walking, knee extensor isometric training, electrical muscle stimulation *Total Volume*: 6 weeks *Progression*: Aerobic intensity progressed to 80% THRmax; isometric training increased to 20 × 5 s; electrical muscle stimulation duration extended to 40 min	Control group (*n* = 12) received no intervention	It improved muscle oxygenation, muscle strength, endurance and reduced fatigue.	Randomized controlled trial.
Wang et al. (2019)	Post-operative patients with breast cancer; Intervention group (*n* = 46) received Baduanjin training + dietary guidance.	*Frequency*: 3 days/week in hospital; 4 days/week at home *Intensity*: Not explicitly stated; Baduanjin is generally low-to-moderate intensity aerobic *Duration*: ~60 min/session (10 min warm-up, 40 min Baduanjin, 10 min relaxation) *Type*: Traditional Baduanjin (8 forms, e.g., “Raise Hands to Regulate Triple Burner,” “Open the Bow,” etc.) *Total Volume*: 7 days/week for 6 months *Progression*: No progression in intensity/difficulty described	Control group (*n* = 40) received routine care and health education	Improved heart rate variability, shoulder joint range of motion, depressive symptoms, and quality of life	Randomized, single-blind controlled trial.
Scott et al. (2020)	Postmenopausal breast cancer patients with impaired VO₂peak; Standard-dose group (*n* = 58), Nonstandard-dose group (*n* = 59) Two groups received exercise prescriptions	*Frequency*: 3–4 sessions/week *Intensity*: Standard-dose: ~70% VO₂peak; Nonstandard-dose: 55 to >95% VO₂peak (variable) *Duration*: Standard-dose: 40 min/session, ~160 min/week; Nonstandard-dose: 20–45 min/session, ~120 min/week *Type*: Treadmill walking *Total Volume*: Standard: ~160 min/week; Nonstandard: ~120 min/week *Progression*: Standard: Fixed after 4 weeks; Nonstandard: Adjusted intensity and duration every 8 weeks per dosing algorithm	The control group (*n* = 57) did not undergo the exercise intervention	Better tolerance to non-linear motion, significantly improved quality of life and reduced fatigue	Parallel-group randomized controlled trial.
Min et al. (2024)	Early-stage postoperative patients with breast cancer; Intervention (*n* = 28): daily home + weekly supervised training	*Frequency*: Daily home exercise; Weekly 20–30 min supervised sessions *Intensity*: Adjusted according to patient’s shoulder ROM and strength recovery *Duration*: 1 month Type: Stretching and resistance training *Total Volume*: 1 session/week (supervised), for 4 weeks *Progression*: Gradual increase in exercise intensity and complexity based on shoulder recovery	Control (*n* = 28): received informational booklet only	The shoulder joint function, physical activity level and quality of life were improved.	Parallel-group, two-arm randomized clinical trial.
Sun et al. (2024)	Post-operative patients with breast cancer; Intervention group (*n* = 72): progressive exercise prescription.	*Frequency*: Aerobic: 3–5×/week ≥30 min; Resistance: 2×/week (≥48 h apart); Flexibility: ≥2–3×/week *Intensity*: Aerobic: moderate intensity (RPE 13–15); Resistance: low intensity (40–50% 1RM) *Duration*: 4 weeks *Type*: Warm-up (joint mobility, marching in place); Aerobic (mindfulness yoga, Baduanjin, aerobic routines, upper limb exercises); Resistance (elastic band curls); Flexibility (shoulder ROM); Cool-down (stretching, marching) *Total Volume*: Aerobic: ≥500–1,000 MET-min/week Progression: Resistance: progressed to moderate intensity (50–70% 1RM); intensity, duration, and frequency adjusted based on tolerance and progression principles	Control group (*n* = 60): routine care	The incidence of upper limb lymphedema was decreased after the operation.	Non-randomized controlled trial

HRR, Heart Rate Reserve; THRmax, Theoretical Maximum Heart Rate; HRmax, Heart Rate maximum; RPE, Borg Rating of Perceived Exertion Scale; 1RM, One-Repetition Maximum; RM, Repetition Maximum; MAP, Maximal Aerobic Power; MET, Metabolic Equivalent of Task; ROM, Range Of Motion; VO₂peak, Volume of Oxygen uptake at Peak exercise; VR, Virtual Reality; 3D, 3-Dimensional.

To systematically analyze the clinical application status and efficacy consistency of core parameters of exercise prescription, this study summarized the intensity, frequency, duration and progression scheme included in the literature. The results are shown in Table 3.

**Table 3:** Exercise prescription parameters analysis for breast cancer patients after surgery.

Parameter category	Strength indicator	Effective range in original study	Number of studies participated in	Efficacy consistency assessment basis
Aerobic exercise intensity	HRR	40–60% HRR (Liu et al., 2021); 60–85% HRR (Kilbreath et al., 2020)	2	Middle: The effective range of the report is wide (40–85%), but the number of studies is small.
	RPE	RPE 9–13 (Jiang et al., 2023); RPE 11–15 (Drozd et al., 2024); RPE 13–15 (Sun et al., 2024)	3	High: All three RPE-based studies reported efficacy in the ‘mild to moderate’ range.
	HRmax	40–60% HRmax (Jiang et al., 2023)	1	Cannot evaluate: Only 1 study used.
Resistance exercise intensity	1-RM	40–50% 1RM → 50–70% 1RM (Liu et al., 2021); 60% 1RM → 80% 1RM (Son et al., 2024); 40%--50% 1RM (Sun et al., 2024)	5	High: All five studies demonstrated effectiveness within the 40–80% 1RM range, with most employing progressive overload.
	RM	10–12 RM (Kilbreath et al., 2020)	1	Cannot evaluate: Only 1 study used.
Progression plan	Strength progress	Increase 5–10% of 1RM every 1–2 weeks (Liu et al., 2021; Sun et al., 2024); Adjust load every 4 weeks (Rasmussen et al., 2022)	7	High: All 7 studies describing progress programs used progressive intensity increases and reported positive effects.
	Duration progress	Add 5–10 min every 1–2 weeks (Jiang et al., 2023; Liu et al., 2021)	2	Middle: The number of studies describing this approach is small, but the direction is consistent.

HRR, Heart Rate Reserve; RPE, Borg Rating of Perceived Exertion Scale; HRmax, Heart Rate maximum; 1RM, One-Repetition Maximum; RM, Repetition Maximum.

Table 3 analysis reveals that resistance exercise intensity (40% ~ 80% 1-RM) and progressive loading schemes demonstrate the highest consistency in effectiveness. For aerobic exercises, the intensity range based on Borg’s subjective fatigue rating (RPE 9 ~ 15) also shows strong consistency. The current primary gap lies in significant heterogeneity regarding the quantification and reporting of core parameters (e.g., intensity, frequency, total exercise volume), coupled with vague descriptions of exercise progression principles, which directly hinders the development of standardized exercise prescription guidelines.

#### 3.4.1 Evaluation of the effectiveness of postoperative exercise prescriptions for breast cancer patients

Evaluating the effectiveness of postoperative exercise prescriptions is a critical component of rehabilitation nursing, as both the scientific rigor and individualization of such prescriptions directly impact recovery outcomes and quality of life. Among these, interventions that combine aerobic and resistance training, incorporate natural daily activities, integrate digital technologies, and utilize traditional Chinese exercise modalities provide patients with more accessible, enjoyable, and personalized exercise experiences. This integration of exercise into everyday life enhances feasibility and patient acceptance, making such approaches more suitable for broad implementation. Detailed findings are presented in Table 4.

**Table 4:** Effects of different exercise intervention models on postoperative rehabilitation outcomes in breast cancer patients.

Exercise intervention model	Studies	Upper limb function (improved/reported)	Lymphedema (improved/reported)	Quality of life (improved/reported)	Adherence (completion/dropout)
combined mode (aerobic + resistance, etc.)	8	7/7	4/4	1/2	Completion rates:>80% (Liu et al., 2021); 80.2% (Dieli-Conwright et al., 2018); 92.9% (Min et al., 2024) Main reasons for discontinuation: treatment side effects (fatigue, pain), personal reasons
Single mode: resistance training	4	3/4	3/3	0/0	Completion rate: 100% (Rasmussen et al., 2022; Son et al., 2024); Main cause of dropout: None
single mode: aerobic training	1	0/0	0/0	0/0	Completion rate: 82% (Scott et al., 2020) Main reason for dropout: Not clearly reported
Traditional physical and mental exercises (Baduanjin, yoga, etc.)	3	2/2	1/1	3/3	Completion rate: 89% (Wang et al., 2019); 93% (Đorđević et al., 2024) Main reasons for dropout: personal reasons/time conflicts, lost to follow-up
Digital/Remote Guidance	3	3/3	2/2	1/2	Completion rate: 92.11% (Jiang et al., 2023; Jin et al., 2018) Main reasons for dropout: lost to follow-up, personal reasons

#### 3.4.2 Effect of different exercise modes on outcome index in postoperative patients with breast cancer

Table 4 demonstrates the effects of different exercise intervention models on upper limb function, lymphedema, quality of life, and adherence in postoperative breast cancer patients. Regarding the impact of exercise modalities on outcome indicators, both combined modalities (aerobic plus resistance training) and single resistance training showed significant positive effects on improving upper limb function and lymphedema (Dieli-Conwright et al., 2018; Esteban-Simon et al., 2023; Kilbreath et al., 2020), establishing them as core measures for enhancing physical function. Notably, two small-sample exploratory studies reported 100% completion rates of resistance training with no participant withdrawals (Rasmussen et al., 2022; Son et al., 2024), preliminarily confirming its safety and acceptability under strict supervision. This provides foundational evidence for the safe and systematic clinical implementation of such training. In terms of improving quality of life and psychological adaptability, traditional mind–body exercises (e.g., Baduanjin, yoga) demonstrated unique advantages (Wang et al., 2019; Xu and Wang, 2021), with all studies reporting positive outcomes. Digital remote guidance has proven to be an effective strategy for improving intervention adherence, with high completion rates (92–94%) and accessibility contributing to enhanced outcomes (Jiang et al., 2023; Jin et al., 2018). Comprehensive analysis indicates that selecting exercise rehabilitation programs for postoperative breast cancer patients requires a holistic approach that considers core rehabilitation goals, individual capabilities, and medical resources to achieve personalized outcomes and maximize benefits.

#### 3.4.3 Factors influencing adherence to exercise prescription for breast cancer

Table 4 reveals variations in adherence across exercise modalities. The combined modalities and digital remote guidance demonstrated higher completion rates (>80 to 94%), while traditional physical and mental exercises also showed strong performance (89–93%). However, treatment withdrawal for combined modalities was primarily attributed to adverse effects (e.g., fatigue, pain) and personal reasons, whereas traditional exercises and digital guidance were mainly discontinued due to personal time conflicts and loss to follow-up. Notably, qualitative research data indicates that some patients still experience lymphedema-related fear during resistance training (Husebø et al., 2015), which correlates with adherence patterns observed in quantitative studies. This finding highlights a critical consideration in designing intervention strategies: treatment-related side effects (fatigue, pain) and specific psychological barriers (e.g., lymphedema fear) serve as major obstacles to exercise adherence, while accessible intervention formats (e.g., digital guidance) and culturally adapted exercise approaches can enhance long-term patient engagement.

#### 3.4.4 Safety of exercise prescription in patients with breast cancer

A limited number of studies have documented mild, transient, and reversible adverse effects, primarily associated with initial exercise loads. These include delayed onset muscle soreness during resistance training, a normal physiological adaptation that typically resolves with continued training (Dieli-Conwright et al., 2018) and reversible fatigue exacerbation in some patients following low-intensity activities during chemotherapy or radiotherapy (Wechsler et al., 2023) Medical staff should accurately identify the nature of the discomfort in the affected limb, distinguish between benign functional adaptation and adverse overload, and dynamically adjust the intensity and duration of exercise based on the patient’s treatment stage and individual response.

#### 3.4.5 Comparison of exercise prescription for Chinese breast cancer patients with international exercise prescription

To gain a deeper understanding of the localization characteristics of postoperative exercise rehabilitation for breast cancer in China, this study conducted a stratified comparison of 6 Chinese studies and 17 international studies. The comparison revealed significant differences in intervention strategies and focal points. In terms of exercise types, Chinese studies showed a marked preference for traditional mind–body exercises such as Baduanjin and the “Eight Shoulder Joint Exercises” based on qigong, whose gentle and continuous nature better aligns with the local cultural perception of rehabilitation, while international studies focused more on modern resistance and aerobic training. In terms of intervention models, Chinese studies integrated “Internet+” and digital technologies, such as WeChat platforms and VR systems, earlier and more extensively, accounting for 50% of the cases, highlighting their infrastructure advantages in digital health applications. In terms of rehabilitation priorities, international studies paid more attention to the impact of exercise on long-term biomedical indicators such as metabolic syndrome and sarcopenic obesity, while Chinese studies focused more on improving upper limb function, preventing lymphedema, and enhancing short-term treatment adherence. The patient showed excellent adherence. The completion rate was between 82 and 94% in the nurse-coordinated and led rehabilitation intervention, and there was a sustained and significant improvement in key rehabilitation outcomes, including upper limb function and lymphedema control (Liu et al., 2021; Xu and Wang, 2021). These differences reflect different medical cultures, resource endowments, and research orientations, suggesting that when formulating rehabilitation plans in China, the acceptance of local culture and the accessibility of existing technical resources must be fully considered.

### 3.5 Postoperative exercise experiences among breast cancer patients

Qualitative studies have explored the multifaceted experiences of breast cancer patients with postoperative exercise. Husebø et al. (2015) described the multidimensional benefits of exercise-including psychological, physiological, and social improvements-through a qualitative approach. Wechsler et al. (2023) focused on patients experiencing cancer-related fatigue, emphasizing personalized strategies for regulating exercise dosage. Drawing on self-regulation theory, Tsai et al. (2018) analyzed factors influencing exercise behavior among cancer survivors. Nielsen et al. (2020) proposed an exercise intervention framework based on mHealth technology tailored to chemotherapy patients. Together, these studies highlight the complexity and individualization of exercise rehabilitation for breast cancer patients, offering valuable insights into clinical nursing practice. Details are summarized in Table 5.

**Table 5:** Postoperative exercise experiences among breast cancer patients.

Theme	Category	Illustrative quote	Number of studies participated in
Positive Impact of Exercise on Physical and Mental Health	Physical health	“I had been down and sorry for myself, thinking life was terrible… Then I realized sitting around feeling sorry would not help. If I went out and did something and thought positively, I’d feel better.” — Husebø et al. (2015)	2 items (Husebø et al., 2015; Wechsler et al., 2023)
		“I felt energized and happy… more alert and ready to take on the day.” — Wechsler et al. (2023)	
	Mental health	“After I finish exercising, I usually feel energized… my mood is definitely better; I feel like my mind works better when I exercise.” — Wechsler et al. (2023)	3 items (Husebø et al., 2015; Tsai et al., 2018; Wechsler et al., 2023)
		“It feels amazing when you realize you have done it—you are sticking with it—and you are part of a study focusing on physical activity. It makes me feel like I’m in control and that things will get better.” — Husebø et al. (2015)	
		“I rewarded myself with a chocolate bar and a piece of cake—and I felt great, no guilt at all.” — Tsai et al. (2018)	
Exploring and Adjusting Exercise Dose	Treatment-related barriers	“I experienced menopausal symptoms during chemo—hot flashes, sweating, and hair loss—which made me feel really uncomfortable. Exercising during hot flashes can feel awful.” — Nielsen et al. (2020)	2 items (Nielsen et al., 2020; Wechsler et al., 2023)
		“I was exhausted for days. Not just the first day—also the second, and third. I did not realize how tired I’d be.” — Wechsler et al. (2023)	
	Goal setting	“I just wanted to become more active. That helped me get back into my routine, more than doing specific exercises.” — Tsai et al. (2018)	3 items (Husebø et al., 2015; Tsai et al., 2018; Wechsler et al., 2023)
		“Participating in this program, going out for walks—I see it as something I’m doing to get better.” — Husebø et al. (2015)	
		“It’s always about what your previous baseline was, and what your new baseline is… It’s frustrating and depressing.” — Wechsler et al. (2023)	
	Finding the right dose	“Every day I wonder: is this too much, or just right?” — Wechsler et al. (2023)	3 items (Husebø et al., 2015; Nielsen et al., 2020; Wechsler et al., 2023)
		“Programs should account for side effects and how patients respond, while encouraging them to stay active. Toward the end of treatment, side effects worsen. Some days rest may be better than exercise.” — Nielsen et al. (2020)	
		“I felt something tight in my arm. After using the resistance band a couple of times, it went away. I still feel a little tightness sometimes, but the exercise helps.” — Husebø et al. (2015)	

#### 3.5.1 Positive effects of exercise on physical and mental health

On the physical health level, one patient stated: *“I had been down and sorry for myself, thinking life was terrible… Then I realized sitting around feeling sorry would not help. If I went out and did something and thought positively, I’d feel better.”* (Husebø et al., 2015), affirming the role of exercise in alleviating negative emotions. Another patient shared: *“I felt energized and happy… more alert and ready to take on the day.”* (Wechsler et al., 2023), highlighting the enhancement of vitality through exercise.

Regarding mental health, one participant described: *“After I finish exercising, I usually feel energized… my mood is definitely better; I feel like my mind works better when I exercise.”* (Wechsler et al., 2023), suggesting potential cognitive benefits from physical activity. Another patient expressed: *“It feels amazing when you realize you have done it—you are sticking with it—and you are part of a study that focuses on physical activity. It makes me feel like I’m in control and that things will get better.”* (Husebø et al., 2015), reflecting an improvement in self-efficacy. In some cases, patients also employed self-reward strategies: *“I rewarded myself with a chocolate bar and a piece of cake—and I felt great, no guilt at all.”* (Tsai et al., 2018).

#### 3.5.2 Variability in postoperative exercise experiences among breast cancer patients

Patients exhibit differing levels of tolerance and responses to treatment-related side effects, resulting in highly individualized exercise experiences. Exercise goals vary across individuals—some patients prefer incorporating natural, everyday physical activities into their routines, while others favor more structured exercise programs. This reflects the personalized nature of exercise needs (Tsai et al., 2018). When attempting to determine an optimal exercise dose, patients often rely on their own perceptions of fatigue and physical response. However, in the absence of professional guidance, this trial-and-error process frequently leads to frustration. Additionally, the available online information does not adequately meet patients’ needs for exercise-related support during chemotherapy, highlighting the urgent need for precise and personalized clinical exercise guidance (Nielsen et al., 2020).

### 3.6 Integrative discovery: the connection between evidence and experience

This study reveals the dynamic process and underlying mechanisms of postoperative exercise prescription from theoretical formulation to clinical practice effectiveness by integrating quantitative and qualitative evidence.

#### 3.6.1 Dual pathways of efficacy: physiological improvement and psychological empowerment

Quantitative results demonstrated that the aerobic-resistance combined training model showed the most significant improvement in upper limb function among breast cancer postoperative patients (see Section 3.4). Qualitative findings provided crucial psychological insights: Participants in this structured program consistently reported enhanced self-control and increased sense of achievement (see Section 3.5). This indicates that the integrated training approach not only delivers multidimensional physiological stimulation but, more importantly, provides patients with continuous “success experiences” through measurable rehabilitation progress, thereby effectively boosting their self-efficacy. The elevated self-efficacy further translates into sustained motivation for exercise and overcoming challenges, creating a virtuous cycle of “functional improvement → psychological reinforcement → behavioral maintenance” in rehabilitation.

#### 3.6.2 The real dilemma of compliance: between self-regulation and symptom burden

Quantitative analysis indicates that treatment-related adverse reactions are one of the primary reasons for patients to discontinue exercise (see 3.4). Qualitative experiences further depict the individualized landscape of this dilemma: patients persistently struggle with symptoms such as fatigue and pain during the process of “exploring appropriate exercise intensity” (see 3.5). The combination of these findings suggests that poor adherence is not solely due to “lack of motivation,” but rather results from the disruption of behavioral regulation caused by the severe and unstable symptom burden during the disease recovery phase, which impairs the patient’s self-regulation capacity. Therefore, the focus of improving adherence should not be limited to education and supervision, but rather on helping patients master more flexible self-regulation strategies to enhance their coping ability during symptom fluctuation periods.

#### 3.6.3 Insights from localization practices: cultural identity enhances intervention affinity

In quantitative studies conducted in China, intervention programs that integrate traditional physical and mental exercises with digital health technologies have demonstrated better patient acceptance and rehabilitation outcomes. Qualitative experiences further reveal that patients value the “harmony of form and spirit” emphasized by such exercise methods, as well as the “convenience” and “sense of companionship” brought by digital tools (see 3.5). This suggests that rehabilitation measures tailored to patients’ cultural backgrounds and health concepts are more likely to stimulate their intrinsic motivation for participation, enhance the affinity and sustainability of interventions, and provide practical evidence for achieving truly “patient-centered” rehabilitation care.

### 3.7 Sensitivity analysis

To ensure the reliability of the qualitative synthesis conclusions in this study, we conducted subgroup analyses based on exercise types, surgical approaches, and intervention duration. Through systematic exclusion of low-quality studies, small-sample studies, and special intervention protocols, we validated the results. The core finding that “structured exercise has rehabilitative benefits for breast cancer postoperative patients” remained consistent across different subgroups and literature subsets after exclusion, demonstrating strong stability. The heterogeneity of existing evidence did not undermine this core conclusion but instead highlighted the need for systematic and standardized development of exercise prescription parameters.

## 4 Discussion

### 4.1 Scientific rigor and standardization of exercise prescriptions

The results of this study show that resistance training has positive effects on the function of the upper limb and lymphedema in breast cancer patients, with the highest evidence strength. The scientifically designed postoperative exercise rehabilitation intervention for breast cancer patients can significantly improve the upper limb motor function of the patients (Leclerc et al., 2017), and at the same time, it will not increase the risk of postoperative lymphedema (Đorđević et al., 2024). This is consistent with the current research conclusions focusing on postoperative exercise rehabilitation for breast cancer (Li et al., 2018), further confirming the core value and evidence-based basis of standardized exercise intervention in the postoperative rehabilitation of breast cancer. But, our results confirm that the scientific rigor and standardization of postoperative exercise prescriptions remain insufficient, as evidenced by the substantial heterogeneity in core parameters (e.g., intensity, frequency) summarized in Table 3. Although traditional Chinese practices such as Baduanjin have shown unique advantages, particularly their integrative mind–body nature that combines meditation, breathing regulation, and structured movements, which aligns well with cultural preferences and provides a holistic approach to recovery (Wang et al., 2019), quantitative analysis of key parameters is still limited. To address these challenges, future research should aim to establish a multidimensional standard system that integrates both objective measurements and subjective assessments. Enhanced digital monitoring and personalized guidance are needed, alongside strengthened evidence-based research on traditional exercise modalities. Long-term follow-up studies and stage-based interventions should also be conducted to develop more scientific, precise, and sustainable exercise prescriptions for breast cancer survivors.

### 4.2 Personalization and adherence in exercise prescription

Postoperative exercise rehabilitation for breast cancer patients faces significant challenges in terms of personalization and adherence (Wechsler et al., 2023). The postoperative exercise compliance of breast cancer patients is constrained by factors such as treatment-related side effects like fatigue, pain, fear-induced lymphedema, and lack of personalized support (Husebø et al., 2015; Wechsler et al., 2023), which is consistent with the existing research conclusions (Wang et al., 2022). This further validates the prevalence of influencing factors for exercise compliance in postoperative breast cancer patients in clinical practice. Current exercise prescriptions often lack a comprehensive and systematic assessment of patients’ health status, functional capacity, and psychological needs, resulting in a poor match between the prescription and individual patient characteristics (Li et al., 2025). First, patients exhibit substantial variability in their exercise experiences (Deng et al., 2021). Qualitative studies (Husebø et al., 2015; Tsai et al., 2018) have confirmed that while exercise can improve mood, enhance self-efficacy, and alleviate physical discomfort, treatment-related side effects and exercise-related fear frequently serve as major barriers to adherence (Nielsen et al., 2020). Second, contradictions exist in intervention strategies. On the one hand, external feedback—such as motivational text messages and goal-oriented tasks—can help maintain exercise behaviors in some patients (Nielsen et al., 2020). On the other hand, some patients prefer autonomy in selecting the type and timing of their activities and may resist overly structured interventions (Tsai et al., 2018). This tension reveals a critical shortcoming of current strategies: they fail to respect individual autonomy and apply a one-size-fits-all approach that does not meet the diverse needs of patients. Regarding the positive effects of “Internet+” digital technology and China traditional exercise on improving postoperative compliance and quality of life for breast cancer patients, the current evidence is of moderate strength. Although the quality of relevant randomized controlled trials is high and shows significant benefits, the number of studies in this field remains relatively limited, and the mechanisms of traditional exercises and the long-term effects of digital technology have not been fully elucidated. More research is needed in the future to consolidate these findings.

To address these issues, the following strategies are recommended:

Before developing an exercise prescription, breast cancer patients should undergo comprehensive health assessments and physical fitness evaluations. The prescription should then be dynamically tailored to match patients’ physical condition, treatment stage, exercise habits, and psychological needs. It is essential to ensure that exercise programs are not only safe and effective but also personalized. During implementation, a balance should be maintained between professional guidance and patient autonomy, with full consideration given to multidimensional factors such as age, physiological function, and individual exercise preferences.

### 4.3 The critical role of multidisciplinary collaboration in exercise rehabilitation for postoperative breast cancer patients

With the continuous advancement of the “integration of medicine and physical activity” model, greater professional competence is expected of nurses in the field of exercise rehabilitation (Zhou et al., 2024). However, many nurses currently lack sufficient knowledge and skills in this area, as well as systematic training in sports medicine, making it difficult for them to accurately assess patients’ exercise capacity and formulate scientifically sound exercise prescriptions (Gao et al., 2020). Furthermore, due to heavy clinical workloads, nurses often have limited time and capacity to conduct detailed follow-up and supervision, which compromises the effectiveness of exercise rehabilitation (Wang et al., 2022). To enhance the role of nurses in postoperative exercise rehabilitation for breast cancer patients, medical institutions should strengthen specialized training programs and promote the development of breast cancer case managers. This will help improve nurses’ competencies in exercise prescription development, injury prevention, and rehabilitation guidance. Effective allocation of nursing human resources is also essential to ensure that nurses or case managers have sufficient time and energy to support patients’ rehabilitation processes. In addition, nurses or case managers should lead the establishment of multidisciplinary exercise rehabilitation teams, fostering close collaboration and communication among nurses, physicians, physical therapists, and mental health professionals. By providing comprehensive, high-quality, and coordinated exercise rehabilitation services, such teams can promote the development of more scientific and personalized approaches to postoperative recovery in breast cancer patients.

### 4.4 Strengths and limitations

This study strictly followed the PRISMA guidelines and the MMAT quality assessment standards, including high-quality evidence-based studies. It integrated quantitative and qualitative research, covered both domestic and international practices, and closely aligned with the “Healthy China 2030” planning outline and clinical needs. The conclusions have clear practical guidance value for the formulation of localized rehabilitation plans. The limitations mainly lie in the existence of language and publication bias risks in the literature inclusion, and significant heterogeneity in the exercise prescription parameters and outcome indicator measurement methods of the included studies. This has to some extent affected the comprehensiveness of the research results and the direct comparability of conclusions among different studies. Future research can further optimize by expanding the language coverage and promoting parameter standardization.

## 5 Conclusion

The “Healthy China 2030” Planning Outline explicitly proposes “promoting the deep integration of medicine and physical activity, improving the chronic disease prevention and rehabilitation service system, and achieving a shift from disease treatment to health management” (Lian et al., 2023). This integrative review, which focuses on postoperative exercise prescriptions for breast cancer, is deeply aligned with this initiative and provides strong support for implementing the national health strategy in the field of breast cancer rehabilitation. The research results show that combining aerobic exercise with resistance training, integrating natural physical activities into daily routines, utilizing digital technology, and adopting exercise therapies rooted in China’s traditional culture, such as Baduanjin and Tai Chi, can effectively improve upper limb function. However, existing exercise prescriptions lack standardization across core components such as exercise type, intensity, frequency, and total volume. Inadequate personalization and poor patient adherence remain major barriers to the effective implementation of exercise rehabilitation. Therefore, we call for the national health authorities to lead the effort in collaborating with experts from rehabilitation medicine, sports science, and nursing to develop a “Postoperative Rehabilitation Exercise Guide for Breast Cancer” tailored to China’s specific conditions. By integrating the principles of “standardization” and “personalization,” we aim to establish an effective localized intervention program. Additionally, training programs should be expanded to improve nurses’ knowledge and skills in exercise rehabilitation, and more breast cancer case managers should be developed. Establishing multidisciplinary rehabilitation teams will be essential for promoting localized, scientific, and precise exercise rehabilitation practices, ultimately improving overall recovery outcomes for breast cancer patients and advancing the goals of the “Healthy China” initiative.

## Data Availability

The original contributions presented in the study are included in the article/Supplementary material, further inquiries can be directed to the corresponding authors.
