# Supplementary material for: Postoperative exercise rehabilitation and patient experience among breast cancer survivors under the “Healthy China” initiative: an integrative review
Source: Front Psychol. 2026 Jan 12;16:1681492. doi: 10.3389/fpsyg.2025.1681492 (PMC12833218; doi:10.3389/fpsyg.2025.1681492)
Supplement: Supplementary file 1 [file Data_Sheet_1.PDF]

Supplementary File 1：search strategy

Database	Retrieval strategy	Number
**PubMed**	("Postoperative Period"[MeSH Terms] OR ("Postoperative Period"[Title/Abstract] OR "Postoperative"[Title/Abstract] OR "Postsurgical"[Title/Abstract] OR "Post-mastectomy"[Title/Abstract])) AND ("Breast Tumor"[Title/Abstract] OR "Breast Cancer"[Title/Abstract] OR "Breast Carcinoma"[Title/Abstract] OR "breast neoplasm"[Title/Abstract] OR "Breast Neoplasms"[MeSH Terms]) AND ("exercise therapy"[MeSH Terms] OR ("exercise therapy"[Title/Abstract] OR "Physical rehabilitation"[Title/Abstract] OR "Exercise intervention"[Title/Abstract] OR "Rehabilitation exercise"[Title/Abstract] OR "Rehabilitation"[Title/Abstract]))	342
**Cochranne**	#1 MeSH descriptor: [Postoperative Period] explode all trees<br>#2 "Postoperative" or "Postsurgical" OR "Post-mastectomy"<br>#3 #1 OR #2<br>#4 MeSH descriptor: [Breast Neoplasms] explode all trees<br>#5 "Breast Tumor" OR "Breast Cancer" OR "Breast Carcinoma" OR "breast neoplasm"<br>#6 #4 OR #5<br>#7 MeSH descriptor: [Exercise Therapy] explode all trees<br>#8 "exercise therapy" OR "Physical rehabilitation" OR "Exercise intervention" OR "Rehabilitation exercise" OR "Rehabilitation"<br>#9 #7 OR #8<br>#10 #3 AND #6 AND #9	55
**Web of Science**	TS="Postoperative Period" OR "Postoperative" OR "Postsurgical" OR "Post-mastectomy" (Topic) AND "Breast Tumor" OR "Breast Cancer" OR "Breast Carcinoma" OR "breast neoplasm" (Topic) AND "exercise therapy" OR "Physical rehabilitation" OR "Exercise intervention" OR "Rehabilitation exercise" OR "Rehabilitation" (Topic)	570

Database	Retrieval strategy	Number
**CINAHL**	XB ("Postoperative Period" OR "Postoperative" OR "Postsurgical" OR "Post-mastectomy") AND XB ("Breast Tumor" OR "Breast Cancer" OR "Breast Carcinoma" OR "breast neoplasm") AND XB ("exercise therapy" OR "Physical rehabilitation" OR "Exercise intervention" OR "Rehabilitation exercise" OR "Rehabilitation")	118
**CNKI (China National Knowledge Infrastructure)**	（篇关摘：乳腺癌 ＋ 乳腺肿瘤(精确)）AND（篇关摘：术后(精确)）AND（篇关摘：运动康复 ＋ 运动治疗 ＋ 功能康复(精确)）	527
**WanFang Data**	摘要:(“乳腺癌”OR“乳腺肿瘤”) and 摘要:(“术后”) and 摘要:(“运动康复”OR“运动治疗”OR“功能康复”)	463
**VIP (VIP Chinese Journal Database)**	摘要=乳腺癌+乳腺肿瘤 AND 摘要=术后 AND 摘要=运动康复+运动治疗+功能康复	295
