# Supplementary material for: Postoperative exercise rehabilitation and patient experience among breast cancer survivors under the “Healthy China” initiative: an integrative review
Source: Front Psychol. 2026 Jan 12;16:1681492. doi: 10.3389/fpsyg.2025.1681492 (PMC12833218; doi:10.3389/fpsyg.2025.1681492)
Supplement: Supplementary file 2 [file Data_Sheet_2.PDF]

Supplementary File 2: Detailed Methodological Quality Appraisal of Included Studies Using the Mixed Methods Appraisal Tool (MMAT), 2018 Version

Author(s)	Study Design	Screening Questions (S1, S2)	Methodological Quality Criteria					Quality Score
**Randomized Controlled Trials (RCTs)**			**1.1**	**1.2**	**1.3**	**1.4**	**1.5**	
Esteban-Simon et al. (2023)	Randomized controlled trial	Y,Y	Y	Y	Y	Y	Y	100%
Jiang et al. (2023)	Randomized controlled trial	Y,Y	Y	Y	Y	Y	Y	100%
Lozano-Lozano et al. (2020)	Randomized controlled trial	Y,Y	Y	Y	Y	Y	Y	100%
Jin et al. (2018)	Randomized controlled trial	Y,Y	Y	Y	Y	Y	Y	100%
Xu and Wang (2021)	Randomized controlled trial	Y,Y	Y	Y	Y	Y	Y	100%
Paolucci et al. (2021)	Randomized controlled trial	Y,Y	Y	Y	Y	Y	Y	100%
Son et al. (2024)	Randomized controlled trial	Y,Y	Y	Y	Y	N	Y	80%
Dieli-Conwright et al. (2018)	Randomized controlled trial	Y,Y	Y	Y	Y	Y	Y	100%
Kilbreath et al. (2020)	Randomized controlled trial	Y,Y	Y	Y	Y	Y	Y	100%
Đorđević et al. (2024)	Randomized controlled trial	Y,Y	Y	Y	Y	Y	Y	100%
Rasmussen et al. (2022)	Randomized controlled trial	Y,Y	Y	Y	Y	N	Y	80%
Hiraoui et al. (2022)	Randomized controlled trial	Y,Y	Y	Y	N	Y	Y	80%
Wang et al. (2019)	Randomized controlled	Y,Y	Y	Y	Y	Y	Y	100%

Author(s)	Study Design	Screening Questions (S1, S2)	Methodological Quality Criteria					Quality Score
	single-blind trial							
Scott et al. (2020)	Randomized controlled trial	Y,Y	Y	Y	Y	Y	Y	100%
Min et al. (2024)	Randomized controlled trial	Y,Y	Y	Y	Y	Y	Y	100%
**Non-Randomized Studies**			**2.1**	**2.2**	**2.3**	**2.4**	**2.5**	
Liu et al. (2021)	Quasi-experimental (non-randomized)	Y,Y	Y	Y	Y	Y	Y	100%
Leclerc et al. (2017)	Non-randomized controlled trial	Y,Y	Y	Y	Y	Y	Y	100%
Drozd et al. (2024)	Retrospective review	Y,Y	Y	Y	Y	Y	Y	100%
(Sun et al., 2024)	Non-randomized controlled trial	Y,Y	Y	Y	Y	Y	Y	100%
**Qualitative Studies**			**3.1**	**3.2**	**3.3**	**3.4**	**3.5**	
Husebø et al. (2015)	Qualitative study	Y,Y	Y	Y	Y	Y	Y	100%
Wechsler et al. (2023)	Qualitative study	Y,Y	Y	Y	Y	Y	Y	100%
Tsai et al. (2018)	Qualitative study	Y,Y	Y	Y	Y	Y	Y	100%
Nielsen et al. (2020)	Qualitative study	Y,Y	Y	Y	Y	Y	Y	100%

Note: According to the MMAT (2018 edition) assessment criteria Part 1: Screening Questions (all studies must answer first) S1. Is there a clear research question? S2. Can the collected data answer the research question? Only if both questions are answered with a "yes" can the subsequent methodological quality evaluation be conducted.

Part 2: Methodological Quality Standards, Category 1: Randomized Controlled Trials 1.1. Is the

randomization procedure appropriate? 1.2. Are the groups comparable at baseline? 1.3. Were all study participants fully assessed for outcome measures? 1.4. Were the outcome assessors unaware of the intervention protocol? (Was blinding implemented?) 1.5. Did the study participants adhere to the assigned intervention protocol?

Category 2: Non-randomized studies 2.1. Do the study subjects represent the target population? 2.2. Are the measured exposure factors (interventions) accurate? 2.3. Is the selection/inclusion method for all study subjects the same? 2.4. Have key confounding factors been considered? 2.5. Were measures taken to avoid interference when measuring outcome indicators during or after the exposure/intervention period?

Category 3: Qualitative Research 3.1. Is the qualitative method appropriate? 3.2. Is the research design based on the research question? 3.3. Is the selection of research subjects appropriate? 3.4. Is the data collection appropriate? 3.5. Is the analysis and interpretation of the data appropriate?

Y=Yes；N=No.
